# Comment on “Improving clinical diagnosis of early-stage cutaneous melanoma based on Raman spectroscopy”

**DOI:** 10.1038/s41416-019-0430-9

**Published:** 2019-03-22

**Authors:** Vincenzo De Giorgi, Federica Scarfì, Alessia Gori, Alessandro Topa, Luciana Trane, Francesca Portelli, Piero Covarelli

**Affiliations:** 1 Department of Dermatology, University of Florence, Florence, Italy; 2 Cancer Research “Attilia Pofferi” Foundation, Pistoia, Italy; 3 Division of Pathological Anatomy, Department of Surgery and Translational Medicine, University of Florence, Florence, Italy; 4 Department of Surgery, University of Perugia, Perugia, Italy

**Keywords:** Melanoma, Cancer screening

We read with great interest the paper by Santos et al. regarding improving the clinical diagnosis of early-stage cutaneous melanoma based on Raman spectroscopy.^1^ However, we disagree with the authors’ conclusions, which state that their diagnostic model based on Raman spectroscopy has enabled greater sensitivity and specificity in melanoma diagnosis, detecting all thin melanomas and reducing the number of unnecessary excisions by more than two-fold compared with the current clinical practice. The main bias in the study’s method is the case series used, which does not allow for the conclusions made by the authors. Namely, the pigmented skin lesions were enroled in the study after a dermatologist performed a clinical assessment and selected lesions that were clinically suspicious for melanoma that had to be excised. Therefore, selection of these lesions was made by a dermatologist and not by the studied model or by use of the Raman method. All this does not represent real clinical practice. Also, because in everyday practice, melanomas are not only pigmented, as in the case studies presented in the paper, but they are also amelanotic, about 10% of cases, which still presents a considerable diagnostic delay compared to other pigmented lesions. Moreover, from this already select series of 222 skin lesions, 48 other lesions were excluded after histopathological examination—some for technical reasons, such as spectral artefacts or equipment failure, and others because they were not melanocytic. We are of the opinion that with all these exclusions and selections do not represent reality and thus, we cannot calculate sensitivity and specificity to compare with real practice to arrive at the same conclusions of the authors. A common opinion is that sensitivity in the clinical diagnosis of melanoma, even made by a specialised dermatologist, is not satisfactory regarding concerns about the risks of false negative diagnosis in practice. Nevertheless, physicians not only excise well-established melanomas but also many equivocal lesions to minimise the risk of leaving a melanoma unexcised. Indeed, only a melanoma left unexcised represents a clinically relevant false negative diagnosis. According to our experience, a melanoma left unexcised does not frequently occur, and this evidence is likely limited to subjects harbouring a clinically “featureless” tumour. In these subjects, the use of new, non-invasive methods for melanoma diagnosis does not seem to have led to a decrease in mortality rates due to melanoma in the past 10 years, but rather to an increase in diagnoses of indolent forms that perhaps may have never progressed. The demonstration that Raman spectroscopy achieves better lesion classification than naked-eye examinations in lesions is only of little reassurance when assessed retrospectively, or in lesions already planned for excision. In our opinion, Raman spectroscopy will certainly occupy an important space in the early diagnosis of melanoma. However, the results presented in this paper are biased by the study including lesions already excised and deemed suspicious by a dermatologist. Moreover, this lesion pre-selection frequently includes many melanomas that are easy to diagnose and which often have an exceedingly high frequency of malignancies within the lesions examined, thus creating an “artificial” diagnostic setting compared to real practice. Only evidence-based studies can provide fully convincing data about the role of Raman spectroscopy in melanoma screening.
